# Negative effects of iodine-based contrast agent on renal function in patients with moderate reduced renal function hospitalized for COVID-19

**DOI:** 10.1186/s12882-021-02469-w

**Published:** 2021-08-31

**Authors:** Anna Kistner, Chen Tamm, Ann Mari Svensson, Mats O. Beckman, Fredrik Strand, Magnus Sköld, Sven Nyrén

**Affiliations:** 1 Medical Radiation Physics and Nuclear Medicine, Karolinska University Hospital, 171 76 Solna, Stockholm, Sweden; 2 Department of Molecular Medicine and Surgery, Karolinska Institutet, Stockholm, Sweden; 3 Department of Radiology, Karolinska University Hospital, Solna, Stockholm, Sweden; 4 Department of Medicine Solna, Karolinska Institutet, Stockholm, Sweden; 5 Department of Respiratory Medicine and Allergy, Karolinska University Hospital, Stockholm, Sweden

**Keywords:** Iodinated contrast, Computed tomography, COVID − 19, P-creatinine, Contrast-induced acute renal failure

## Abstract

**Background:**

Kidney disease and renal failure are associated with hospital deaths in patients with COVID − 19. We aimed to test if contrast enhancement affects short-term renal function in hospitalized COVID − 19 patients.

**Methods:**

Plasma creatinine (P-creatinine) was measured on the day of computed tomography (CT) and 24 h, 48 h, and 4–10 days after CT. Contrast-enhanced (*n* = 142) and unenhanced (*n* = 24) groups were subdivided, based on estimated glomerular filtration rates (eGFR), > 60 and ≤ 60 ml/min/1.73 m^2^. Contrast-induced acute renal failure (CI-AKI) was defined as ≥27 μmol/L increase or a > 50% rise in P-creatinine from CT or initiation of renal replacement therapy during follow-up. Patients with renal replacement therapy were studied separately. We evaluated factors associated with a > 50% rise in P-creatinine at 48 h and at 4–10 days after contrast-enhanced CT.

**Results:**

Median P-creatinine at 24–48 h and days 4–10 post-CT in patients with eGFR> 60 and eGFR≥30–60 in contrast-enhanced and unenhanced groups did not differ from basal values. CI-AKI was observed at 48 h and at 4–10 days post contrast administration in 24 and 36% (*n* = 5/14) of patients with eGFR≥30–60. Corresponding figures in the eGFR> 60 contrast-enhanced CT group were 5 and 5% respectively, (*p* < 0.037 and *p* < 0.001, Pearson *χ*^*2*^ test). In the former group, four of the five patients died within 30 days. Odds ratio analysis showed that an eGFR≥30–60 and 30-day mortality were associated with CK-AKI both at 48 h and 4–10 days after contrast-enhanced CT.

**Conclusion:**

Patients with COVID − 19 and eGFR≥30–60 had a high frequency of CK-AKI at 48 h and at 4–10 days after contrast administration, which was associated with increased 30-day mortality. For patients with eGFR≥30–60, we recommend strict indications are practiced for contrast-enhanced CT. Contrast-enhanced CT had a modest effect in patients with eGFR> 60.

## Introduction

Iodine-based contrast agents for intravascular use may have a negative effect on kidney function, particularly in previously compromised kidneys [1]. Contrast-induced acute kidney injury (CI-AKI) has been defined as an increase in plasma (P)-creatinine measured 2–3 days after computed tomography (CT) [2]. CI-AKI is defined as an increase in P-creatinine ≥27 μmol/L or > 50% rise in P-creatinine above the value on the day of CT [2]. A previous definition used a P-creatinine increase of 44 μmol/L or above as diagnostic [3]. A recent guideline recommends a lower limit [2]. Patient related factors are known to affect the risk; most importantly kidney function, which is usually expressed as, estimated glomerular filtration rate (eGFR). A value < 60 ml/min/1.73m^2^ has been used to define a somewhat higher risk. eGFR < 30 ml/min/1.73 m^2^ indicates severe renal failure and is considered a risk factor for CI-AKI [1, 2]. The incidence of CI-AKI was shown to range from 0 to 24%, with the highest risk in diabetic nephropathy [4]. Other background factors of importance are kidney surgery, proteinuria and hypertension [2]. It is also well known that severity of present disease increases the risk substantially [5].

Kidney disease and renal failure were associated with hospital death in COVID-19 [4]. Thromboembolic diseases, including pulmonary embolism (PE), are seen with a high frequency in COVID-19 [6, 7]. The primary method for confirming pulmonary embolism is CT angiography [8]. Suspected PE was the dominating indication for CT angiography in our study. These observations raised the question of whether patients with COVID-19 might be more susceptible to the harmful effects of iodinated contrast material [9]. This possibility could influence risk assessments whether a CT angiogram is performed in COVID-19. We hypothesized that, in patients with COVID-19 stratified according to eGFR, the administration of contrast agent in patients with a moderate renal impairment (eGFR≤60 ml/min/1.73 m^2^) would increase P-creatinine and reduce eGFR to a greater extent than an unenhanced CT.

## Methods

This retrospective study was approved by the Swedish Ethical Review authority, (Dnr 2020–01882), and informed consent was waived. From March 19 to May 31, 2020, we included all patients with a PCR test positive for SARS-CoV-2 that were referred to thoracic or abdominal CT at our hospital.

### Measurements

We collected P- creatinine measured on the day of before CT and 24 h, 48 h, and 4–10 days after CT, when available. The collection of P-creatinine values at days 4–10, a little longer interval than normal [2] was performed as a way to study if P-creatinine reacted differently in this patient group after contrast injection but also as a double-follow-up. P-creatinine (anticoagulated with Li-heparin) was analyzed with an enzymatic photometric method (Cobas®, Roche Diagnostics, GmbH, Mannheim, Germany). The reference values were < 100 μmol/L for men and < 90 μmol/L for women. Pre- COVID − 19 levels of P-creatinine were retrieved from patient records up to 2 years before the current hospitalization. These values were collected in order to compare renal function before COVID-19 as this might affect outcome.

eGFR was calculated according to the chronic kidney disease epidemiology collaboration (CKD-EPI) formula [2]. An eGFR ≥90 ml/min/1.73 m^2^ was taken as normal, eGFR values 30–60 indicated moderate reduced renal function and values below 30 indicated severe renal failure [2]. CI-AKI was defined as an increase in P-creatinine ≥27 μmol/L or > 50% rise in P-creatinine above the value on the day of CT [2].

CT was performed with a 256-slice multi-detector Revolution CT (GE Healthcare, Wauwatosa, Wisconsin, USA). Of the 142 patients receiving contrast administration at CT in our study, 115 subjects were investigated with CT angiography of the chest for suspected PE. According to standardized protocol the legitimate total contrast injection was 50 ml, which was slightly higher (70 ml) in adipose patients (> 80 kg). Nevertheless the standard contrast dose received at CT was 60 ml. The contrast agent (Omnipaque 350 mg/ml, Iohexol, GE Healthcare, Wauwatosa, Wisconsin, USA) was injected at a flow rate of 5 ml/s. Remaining patients (*n* = 27) performed CT angiography and CT of the abdomen in venous phase with personalized and higher contrast doses.

We collected data on patient age, sex, weight, height, body mass index (BMI), anti-thrombotic treatment, co-morbidities, C-reactive protein (CRP; reference value < 5.0 mg/l) measured at the time of the CT scan, contrast dose, intensive care unit (ICU) stay and 30-day mortality. CRP was determined with an immuno-turbidimetric analysis (Cobas®, Roche Diagnostics, GmbH, Mannheim, Germany).

### Study groups

**Fig. 1:**
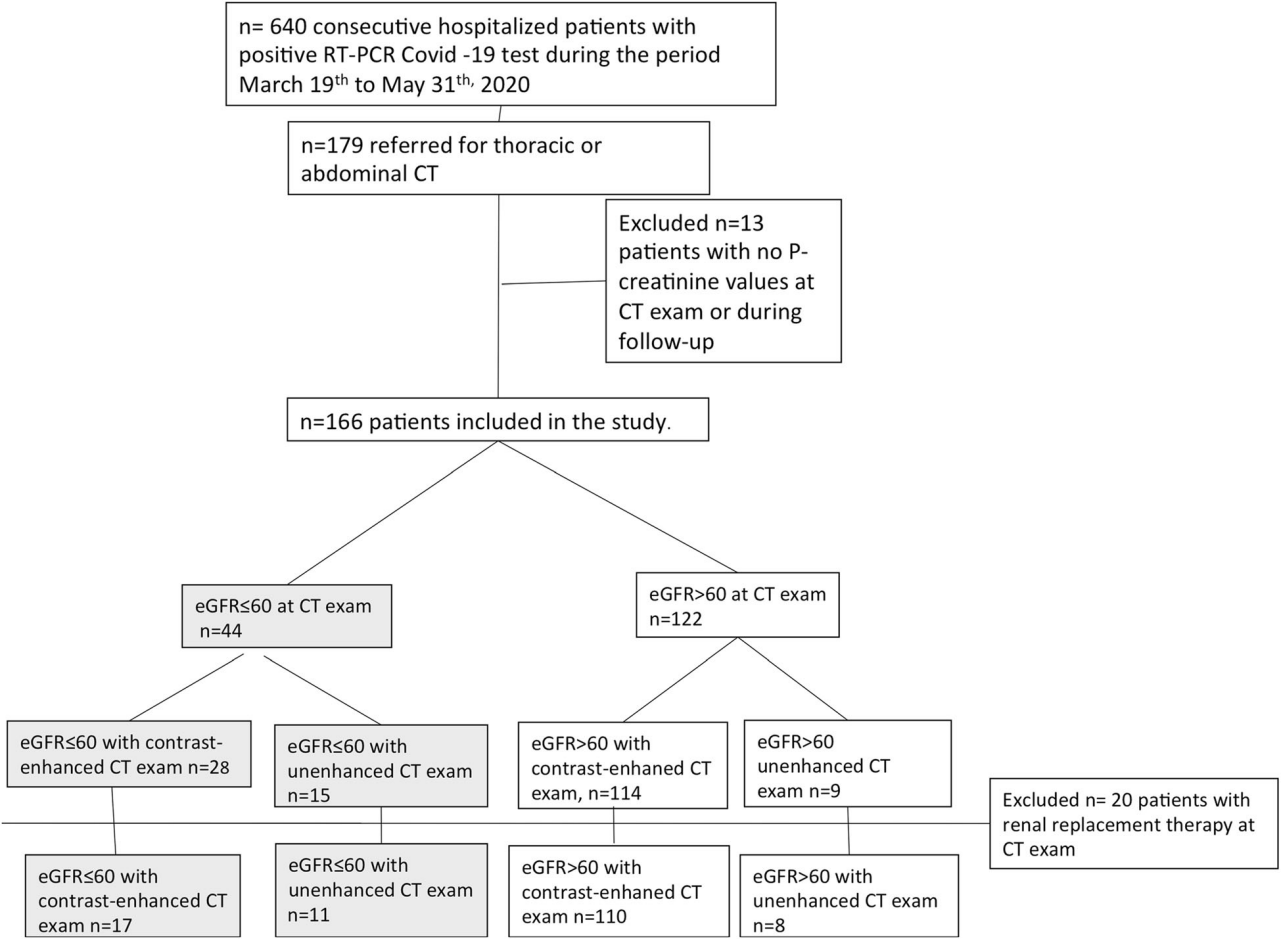
Flowchart shows the patient selection process. CT: computed tomography; eGFR: estimated glomerular filtration rate

The patient selection process is shown in Fig. 1. During the study period, 166 patients met the inclusion criteria. Among these, seven patients were included twice, because they underwent CT scans with and without contrast-enhancement. No patient underwent a repeated contrast-enhanced or unenhanced CT scan.

Patients were divided into subgroups depending on renal function. The subgroups were defined as an eGFR > 60 ml/min/1.73 m^2^ (eGFR> 60) or an eGFR ≤60 ml/min/1.73 m^2^ (eGFR≤60)*.* Patients on renal replacement therapy at CT (*n* = 20) were analyzed separately.

### Statistics

Anthropometric and laboratory data are presented as median and interquartile range (IQR, Table 1). Student t-test, analysis of variance (ANOVA), post-hoc Fischer’s test, and Pearson’s chi-squared (*χ*^*2*^)– test were used for comparisons of categorical variables. Logistic regression analyses were used to calculate the odds ratio (OR) and 95% confidence interval (CI) (Table 2). Odds ratios for a > 50% rise in P-creatinine at 48 h and at 4–10 days after contrast-enhanced CT for variables of interest are presented in Table 2. Median values of certain parameters (P-creatinine at CT, dose agent/ weight and age) for the whole cohort were labeled as cut–off values for odds ratio calculation. A *P*-value < 0.05 was considered significant. All statistical analyses were performed with Statistical Stat Soft, version 10.

**Table 1:** Demographics, laboratory data, and outcome in patients with COVID-19, grouped by eGFR > 60 or ≤ 60 ml/min × 1.73 m^2^ with or without contrast-enhanced CT

(a) Patients without RRT at CT	1. eGFR≥30–60 contrast-enhaned CT (*n* = 17)	2. eGFR> 60 contrast-enhaned CT (*n* = 110)	3. eGFR≤60 unenhanced CT (*n =* 11)	4. eGFR> 60 unenhanced CT (*n* = 8)	*p*-value*
Demographics					
Men/women nr subjects)	14/3	77/33	7/4	6/2	0.65
Age (years)	65 (61–76)	58 (49–64)	59 (51–68)	64 (50–70)	*0.029*
Weight (kg)	75 (72–87)	80 (72–93)	74 (61–87)	79 (64–90)	0.53
BMI (kg/m^2^)	25 (23–30)	28 (24–31)	25 (21–29)	25 (21–27)	0.19
Thromboembolic treatment					
24 h before CT exam (%)(*n* = no/yes), *n* = 144	76 (*n* = 4/*n* = 13)	76 (*n* = 26/ *n* = 84)	89 (*n =* 1/*n =* 8)	88 (*n =* 1/*n =* 7)	0.75
Co-morbidities					
Renal disease *n =* 144	25 (4/12)	0 (0/109)	55 (6/5)	13 (1/7)	*< 0.001*
Hypertension *n* = 141	60 (9/6)	36 (38/68)	64 (7/4)	13 (1/7)	*0.037*
Heart failure *n =* 144	13 (2/14)	5 (5/104)	18 (2/9)	13 (1/7)	0.24
Type 2 diabetes *n* = 143	24 (4/12)	20 (22/86)	18 (2/9)	25 (2/6)	0.96
Laboratory data					
P-creatinine at CT, *n* = 146	130 (121–153)	61 (48–76)	163 (132–201)	65 (45–80)	*1* vs *3 < 0.001/ 2* vs *3 p < 0.001/2* vs *4 0.85/3* vs *4 p < 0.001*
P-creatinine 24 h after CT exam, *n* = 127	130 (118–186)	61 (49–74)	136 (105–151)	63 (43–89)	*1* vs *3 0.97/ 2* vs *4 0.97*
P-creatinine 48 h after CT exam, *n* = 128	130 (111–192)	62 (50–73)	123 (112–159)	69 (54–74)	*1* vs *3 0.46/ 2* vs *4 ns 0.95*
P-creatinine 4–10 days after CT exam, *n =* 110	129 (77–197) (*n =* 14)	62 (48–75) (*n* = 78)	85 (70–124) (*n* = 9)	64 (53–79) (*n* = 9)	*1* vs *3 0.09/ 2* vs *4 0.59*
P-creatinine rise > 26.5 μmol/L or > 50% 48 h after CT exam *n =* 128	24 (*n* = 3/*n =* 14)	5 (*n =* 5/*n* = 92)	20 (*n =* 2/*n =* 8)	0 (*n =* 5)	*0.037* †
P-creatinine rise > 50% at days 4–10 after CT exam and/or RRT initiated, *n =* 110	36 (*n* = 5/*n =* 9)	5 (*n =* 4/*n* = 75)	0 (*n =* 9)	0 (*n =* 8)	*< 0.001* †
CRP at CT scan (mg/L), *n* = 126	186 (71–292)	71 (26–163)	186 (74–257)	96 (42–197)	*0.005, 1* vs *2 p < 0.01, 1* vs *4 p < 0.05*
Total contrast dose (ml), *n =* 111	60 (50–100) (*n =* 15)	60 (60–70) (*n =* 96)	n.a.	n.a.	0.96
Contrast dose (ml/kg), *n* = 98	0.80 (0.69–1.22)	0.76 (0.65–0.94)	n.a.	n.a.	0.58
Outcome data					
ICU support at CT exam *n =* 144	56 (*n =* 9/*n =* 7)	23 (*n =* 25/*n =* 84)	18 (*n =* 2/*n =* 9)	0 (*n* = 0/*n* = 8)	*< 0.01* †
30-day mortality *n =* 146	53 (*n =* 9/*n =* 8)	11 (*n =* 12/*n =* 98)	18 (*n =* 2/*n =* 9)	13 (*n =* 1/*n =* 7)	*< 0.001* †
(b) Patients without RRT at CT and eGFR≥30–60 CE	1. P-creatinine rise > 50% at 4–10 days after CT exam and/or RRT initiated (*n =* 5)	2. P-creatinine rise < 50% at 4–10 days after CT exam (*n =* 9)	*p*-value*		
Demographics					
Age	68 (65–76)	64 (54–70)	0.55		
Weight	72 (64–75)	76 (79–86)	0.21		
Laboratory data					
Pre-Covid P-creatinine	97 (82–106)	97 (77–114)	0.95		
P-creatinine at CT	129 (124–130)	143 (118–155)	0.49		
P-creatinine at 24 h	143 (126–196)	123 (116–139)	0.31		
P-creatinine at 48 h	163 (138–196)	108 (103–126)	0.13		
P-creatinine at 4–10 days	207 (197–237)	88 (68–118)	< 0.001		
CRP in mg/L	295 (274–331)	136 (59–221)	0.06		
Outcome data					
30-day mortality	80 (4/1)	33 (3/6)	0.09 †		
(c) Patients with existing RRT at contrast-enhanced CT vs. the entire unenhanced CT group	1. Contrast-enhaned CT and RRT (*n =* 15)	3. Unenhanced CT (*n =* 24, including 19 without and 5 with RRT)	*p*-value*		
Demographics					
Men/women (nr of subjects)	13/2	18/6	0.37		
Age (years)	59 (55–64)	62 (48–67)	0.65		
Weight (kg)	85 (70–100)	76 (68–90)	0.16		
BMI (kg/m^2^)	28 (24–32)	25 (23–27)	0.08		
Thromboembolic treatment 24 h before CT exam (%)(*n =* no/yes), *n =* 37	87 (*n =* 2/*n =* 13)	86 (*n =* 3/*n =* 19)	0.98		
Co-morbidities					
Renal disease	7 (1/14)	30 (7/17)	0.08		
Hypertension	36 (5/9)	43 (10/13)	0.64		
Heart failure	0 (0/15)	13 (3/20)	0.14		
Type 2 diabetes	13 (2/13)	26 (6/17)	0.35		
Laboratory data					
P-creatinine, *n =* 39	161 (130–232)	129 (80–187)	0.68		
P-creatinine 24 h after CT exam, *n* = 34	151 (132–219)	108 (86–151)	0.47		
P-creatinine 48 h after CT exam, *n* = 33	127 (105–175)	117(74–159)	0.69		
P-creatinine 4–10 days after CT exam, *n =* 33	188 (67–358) (*n =* 14)	82 (62–131) (*n* = 21)	0.12		
P-creatinine rise 26.5 μmol/L or > 50% 48 h after CT exam, *n* = 33	29 (*n* = 4/*n* = 10)	11 (*n* = 2/17)	0.18 †		
P-creatinine rise > 50% at days 4–10 after CT (%), *n =* 33	36 (*n =* 5/*n =* 9)	5 (*n =* 1/20)	*0.017* †		
CRP at CT scan (mg/L)	101 (54–190)	127 (74–204)	0.70		
Total contrast dose (ml), *n =* 14	110 (60–129)	n.a.			
Contrast dose (ml/kg), *n =* 11	1.10 (0.69–1.43)	n.a.			
Outcome data					
ICU support at CT exam, *n =* 39	100 (*n =* 15/*n =* 0)	25 (*n =* 6/*n =* 18)	*< 0.001* †		
30-day mortality, *n =* 39	40 (*n =* 6/*n =* 9)	21 (*n =* 5/*n =* 19)	0.20 †		

Values are presented as the median (interquartile range). Comorbidities, and outcome data are expressed as %, (affected subjects / total subjects in each group). Statistics are based on: *analysis of variance (ANOVA) with post-hoc Fisher’s test, or t-test, or ^†^Pearson *χ* ^2^ test.

*BMI* body mass index, *CRP* C-reactive protein, *CT* computed tomography, *eGFR* estimated glomerular filtration rate (ml/min/1.73 m^2^), *ICU* intensive care unit, *n* number of subjects, *n.a.* not applicable, *non-CE* CT without CE, *P-creatinine* plasma creatinine in μmol/L, *RRT* renal replacement therapy

**Table 2:** Linear regression analysis of variables associated with a > 50% rise in P-creatinine at 48 h or 4–10 days after contrast-enhanced CT

Variable^a^	Nr subjects at 48 h / Nr subjects at 4–10 days after CT	Above or below median value	P-creatinine rise 26.5 μmol/L or > 50% at 48 h after CT, OR (95% CI)	*p*-value	P-creatinine rise 26.5 μmol/L or > 50% and/or RRT initiation at 4–10 days after CT, OR (95% CI)	*p*-value
Male sex	114/93		1.2 (0.2–6)	0.86	3.0 (0.3–26)	0.25
Age (years)	114/102	> 59	2.2 (0.5–9.4)	0.27	4.7 (0.9–24)	0.042
Thromboembolic treatment	114/93		0.59 (0.1–2.6)	0.49	0.95 (0.2–5)	0.96
Renal disease	112/91		4.2 (0.4–46)	0.29	6.8 (0.5–90)	0.19
Type 2 diabetes	111/90		3.3 (0.8–14)	0.11	0.8 (0.1–7)	0.80
P-creatinine (μmol/L) at CT	114/93	> 67	1.7 (0.4–6.7)	0.46	2.4 (0.6–11)	0.22
CRP at CT scan (mg/L)	113/92	> 93	3.8 (0.7–19)	0.08	6.8 (0.8–60)	0.039
Dose/weight (ml/kg)	89/70	> 0.78	0.68 (0.14–3.3)	0.63	0.9 (0.2–3.9)	0.86
eGFR≥30–60 and contrast-enhanced CT	114/93		5.7 (1.3–24)	0.018	10.4 (2.3–47)	0.002
ICU care	113/92		2.0 (0.5–8)	0.34	5.7 (1.2–27)	0.023
30-day mortality	114/93		12 (2.7–54)	< 0.001	16 (3–79)	< 0.001
Renal replacement therapy at CT^b^	128/106		4.7 (1.2–18)	0.037	5.2 (1.4–19)	0.017

^a^ Patients performing contrast-enhanced CT, excluding subjects on renal replacement therapy.

^b^ Including patients on renal replacement therapy.

*CI* confidence interval, *CRP* C-reactive protein, *CT* computed tomography, *eGFR* estimated glomerular filtration rate (ml/min × 1.73 m^2^), *h* hours, *ICU* intensive care unit, *OR* odds ratio, *Nr* Number, *P-creatinine* plasma creatinine, *RRT* renal replacement therapy

## Results

### Patients with eGFR > 60 ml/min/1.73 m^2^ without existing renal replacement therapy examined with contrast-enhancement

**Fig. 2:**
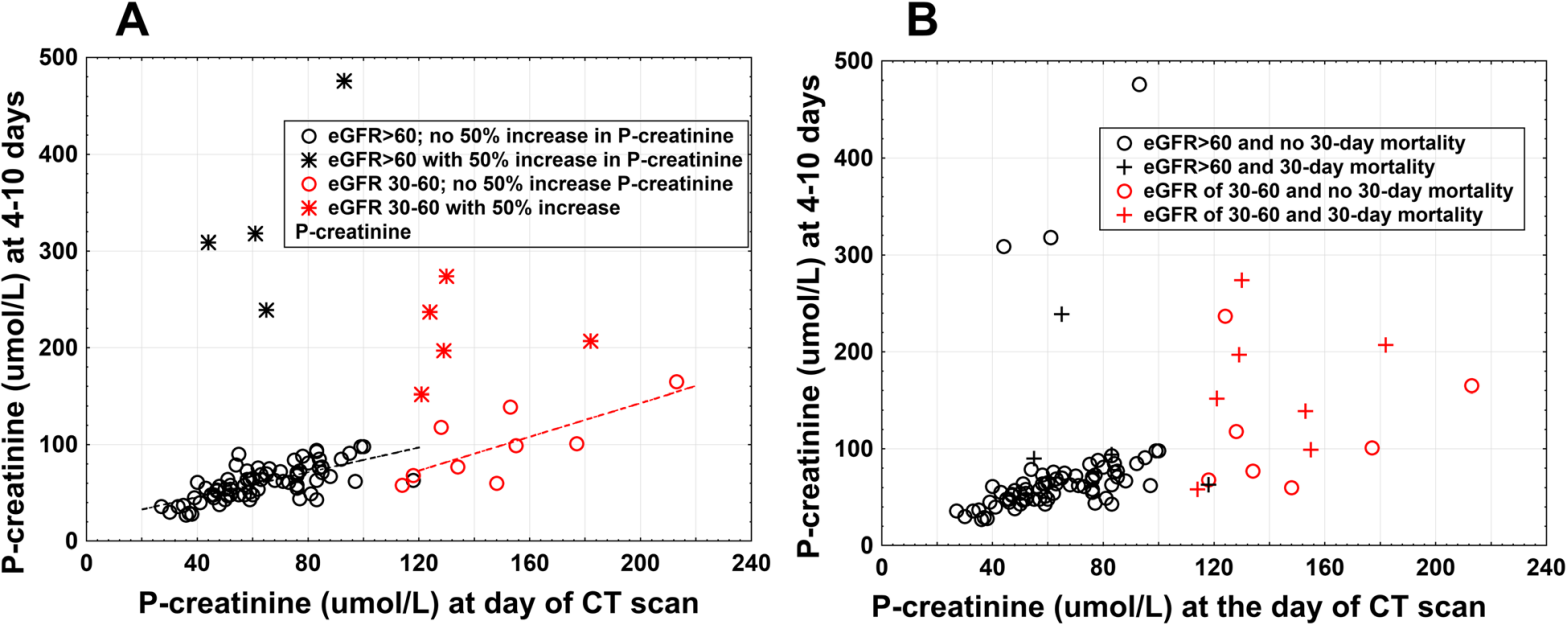
Outcomes, based on eGFR, in patients hospitalized with Covid-19 that underwent contrast-enhanced CT. P-creatinine values (μmol/L) at the time of CT are plotted relative to P-creatinine values at 4–10 days after CT. Patients with renal replacement therapy at the time of CT were excluded. **(a)** eGFR and a contrast-enhanced induced rise in P-creatinine. Black unfilled circles or stars represent patients with an eGFR> 60 ml/min/1.73 m^2^ at CT (*n* = 79). Black stars indicate patients with a > 50% rise in P-creatinine at 4–10 days (*n =* 4). Black unfilled circles represent patients without a > 50% rise in P-creatinine or renal replacement therapy initiation (*n* = 75). The dashed black line shows the correlation between P-creatinine at CT and P-creatinine at 4–10 days; (*r =* 0.71, *p <* 0.001). Red unfilled circles or stars represent patients with an eGFR of 30–60 ml/min/1.73 m^2^ (*n =* 14). Red stars indicate patients with a > 50% rise in P-creatinine or an renal replacement therapy initiation (*n =* 5). Red unfilled circles represent patients without a > 50% rise in P-creatinine or an renal replacement therapy initiation (*n =* 9). The dashed red line shows the correlation between P-creatinine at CT and P-creatinine at 4–10 days (*r =* 0.74, *p* = 0.024). **(b)** eGFR and 30-day mortality. Black unfilled circles and crosses represent patients with an eGFR> 60 ml/min/ 1.73 m^2^. Black unfilled circles represent patients that did not die within 30 days after CT. Black crosses represent patients that died within 30 days after the CT. Red unfilled circles and crosses represent patients with an eGFR of 30–60 ml/min/1.73 m^2^. Red unfilled circles represent patients that did not die within 30 days of the CT. Red crosses represent patients that died within 30 days after the CT. CT: computed tomography; eGFR: estimated glomerular filtration rate; P-creatinine: plasma creatinine

Median P-creatinine at of the first CT scan was 61 μmol/L (IQR 48–76) in the contrast-enhanced CT group (110 patients), while in the unenhanced CT group (8 patients) it was 65 (45–80) μmol/L (*p =* ns, Table 1a). At 48 h median (IQR) P-creatinine was 62 μmol/L (50–73) in the contrast-enhanced group vs 69 μmol/L (54–74) in the unenhanced group (ns, analysis of variances, (ANOVA), Fischer’s post hoc test, Table 1a). Corresponding figures at 4–10 days were 62 μmol/L (48–75) vs 64 μmol/L (53–79) (*p =* ns, Table 1a). However, in the contrast-enhanced CT group at 48 h, five of 97 patients studied and at 4–10 days, four of 78 patients studied showed an increase in P-creatinine above definition for CI-AKI (Table 1a, Fig. 2a).

At 48 h, the P-creatinine in three out of four of these patients was already above definition for CI-AKI. Two of these patients had renal replacement therapy on day 7. In three of the four patients with a > 50% increase in P-creatinine after 4–10 days, the median (IQR) pre-COVID-19 P-creatinine level was 83 μmol/L (65–104).

Six patients died before the 4–10-day follow-up. Of these patients, one had a 200% rise in P-creatinine at 48 h, not seen in the other five patients.

### Patients with eGFR ≥30–60 ml/min/1.73 m^2^ without existing renal replacement therapy examined with contrast-enhancement

In the contrast-enhanced CT group the median (IQR) P-creatinine was 130 μmol/L (121–153) (*n* = 17 patients; Table 1a). This value remained unchanged at 24–48 h, and at 4–10 days after contrast agent exposure for the whole group (Table 1a). However, at 48 h, the incidence of a P-creatinine rise above definition for CI-AKI was observed in 24% vs 5% in the contrast-enhanced CT eGFR> 60 group (*p* = 0.037, Pearsons *χ*^*2*^ test, Table 1a). Five out of 14 patients that remained at follow-up days 4–10 showed a > 50% P-creatinine increase, including the initiation of renal replacement therapy in two cases (Fig. 2a, Table 1a). This incidence was higher than the figures observed in the contrast-enhanced CT eGFR> 60 group (36% vs 5%, *p* < 0.001, Table 1a) and also compared with the eGFR< 60 group not receiving contrast agent (36% vs 0%, *p* = 0.043, Pearsons *χ*^*2*^ -test). These five patients had initial P-creatinine values of 121, 124, 129, 130, and 182 μmol/L (Fig. 2a), and median (IQR) pre-COVID P-creatinine of 97 μmol/L (82–106). Three of these patients had a pre-COVID eGFR < 60 ml/min/1.73 m^2^. These five patients did not seem to differ in pre-COVID P-creatinine compared with other patients in the contrast-enhanced CT ≥ 30–60 group (Table 1b). Four of these five patients died within 30 days of the contrast-enhanced CT (Fig. 2b) and in total, 30-day mortality was 53% in the whole group. Two of these patients died before follow up at 4–10 days. Of the two patients that died before follow-up, one showed a rise in P-creatinine above definition for CI-AKI at 24 h.

A higher pre-COVID-19 prevalence of renal disease and hypertension was seen in the eGFR≥30–60 contrast-enhanced CT group compared with the eGFR> 60 contrast-enhanced CT group (Table 1a). None of contrast-enhanced CT patients with eGFR ≤60 had an eGFR< 30 ml/min/1.73 m^2^.

### Patients with eGFR ≤60 ml/min/1.73 m^2^ without existing renal replacement therapy examined without contrast-enhancement

In the unenhanced CT group, among the 11 patients with eGFR≤60, the initial median (IQR) P-creatinine level was 163 μmol/L (132–201), which was significantly higher than the levels observed in the other groups (Table 1a). In this group, the median P-creatinine level fell progressively during follow-up (Table 1a). At 4–10 days after CT, none of nine patients showed a 50% increase in P-creatinine. 30-day mortality was 18% compared with 53% in the eGFR ≥30–60 contrast-enhanced CT group (*p* = 0.07, Pearsons *χ*^*2*^ - test). Two of unenhanced CT patients with eGFR ≤60 had an eGFR< 30 ml/min × 1.73 m^2^.

In the unenhanced CT group, no patient received renal replacement therapy after CT during the study period.

### Patients with existing renal replacement therapy examined with contrast enhancement

This group (*n* = 15) received higher doses of contrast agent than patients not taking renal replacement therapy (median doses (IQR): 110 ml (60–129) vs. 60 ml (60–70); *p* < 0.001). The median (IQR) P-creatinine levels in this group declined from 161 μmol/L (130–232) at baseline to 127 μmol/L (105–175) at 48 h; then it rose to 188 μmol/L (67–358) at 4–10 days after contrast agent exposure (not significantly different from baseline; Table 1c).

After 4–10 days, we observed a > 50% increase in P-creatinine in five out of remaining 14 patients in the renal replacement therapy with contrast-enhanced CT group and only one out of 21 patients in the non-renal replacement therapy unenhanced CT group (36% vs. 5%, *p* = 0.017, Pearsons *χ*^*2*^-test, Table 1b). Among the 24 patients that received an unenhanced CT during the study period, five were taking renal replacement therapy at the time of the CT (5 patients included in Table 1c).

### Odds ratio analysis

Odds ratio analyses indicated that a > 50% rise in P-creatinine 48 h after contrast agent administration associated with eGFR≥30–60 and contrast-enhanced CT (*p* = 0.018), with 30-day mortality (*p <* 0.001) and with renal replacement therapy at CT (*p* = 0.037) but not with age, male gender or contrast agent dose/weight (Table 2). At 4–10 days, a > 50% rise in P-creatinine was aside from eGFR≤30–60 and contrast-enhanced CT, 30-day mortality and renal replacement therapy at CT also associated with advancing age (*p* = 0.042), ICU stay (*p* = 0.023) and increased CRP at CT (*p* = 0.039) but not with male gender (Table 2).

## Discussion

We hypothesized that the administration of iodine-based contrast agent to patients with COVID − 19 would affect P-creatinine and renal function. In the present study, in all subgroups of renal function, small changes were seen in median values 24–48 h after contrast agent exposure. However, our findings indicated that contrast agent administration had a negative effect on renal function at 48 h in 24% of patients and at 4–10 days in 36% of patients with moderate renal failure and eGFR 30–60 ml/min/1.73m^2^. Corresponding figures observed in patients with eGFR > 60 and contrast-enhancement were significantly lower, 5%. Not surprisingly mortality was higher in contrast-enhanced patients with eGFR of 30–60 compared with those with eGFR above 60 (53% vs 11%). More patients with eGFR between 30 and 60 given contrast agent developed CI-AKI compared to those not receiving contrast agent (24–36% vs 0%). Patients in the unenhanced CT group with eGFR≤60 had a median CRP of 186 mg/L at CT scan (compared to the median in the entire group 93 mg/L) and significantly higher median P-creatinine at CT scan compared with the eGFR≥30–60 contrast-enhanced CT group (163 vs 130 μmol/L, *p <* 0.001). Despite this, 30-day mortality showed a tendency to be lower in the unenhanced CT group. These findings indicate that CI-AKI seems to have identified a vulnerable group of COVID-19 patients.

The findings related to renal replacement therapy are difficult to interpret without detailed knowledge of the renal replacement therapy intensity or diuresis. Not surprisingly, renal failure after contrast-enhanced CT was associated with an ICU stay day 4–10 (*p* = 0.023) and with 30-day mortality (*p <* 0.001).

The peak P-creatinine is typically observed 2 to 5 days after a contrast medium injection [10]. The risk factors associated with acute kidney injury after an iodinated contrast injection includes advanced age, hypovolemia and dehydration, type 2 diabetes, and previously impaired kidney function [11]. Chronic kidney disease was proposed to be the strongest risk factor associated with developing a contrast-associated acute kidney injury [12]. However, Barrios-Lopez et al. reported, before the COVID − 19 era, that the incidence of CI-AKI was only 1% in patients with chronic renal disease and eGFR 30–60 ml/min × 1.73 m^2^ [10].

Moreover, critically ill patients are at increased risk of CI-AKI. In 2011, Hoste et al. reported that one out of six patients in the ICU developed CI-AKI [5]. However, in a recent large meta-analysis with a control group, acute kidney injury was not associated with the administration of iodinated contrast material [13].

Deep vein thrombosis was found in 40% of autopsies in 80 cases with COVID-19 in Germany [14]. COVID − 19 was associated with an increased risk of thrombosis in both the micro- and macrovasculature [15]. Acute kidney injury is a complication associated with severe COVID-19 infections [16]. At authopsies COVID-19 patients had widespread thrombosis and microthrombis in the lungs [17]. One may speculate that COVID-19 patients exhibit similar microvascular damage in the kidney. Furthermore, renal tubule cells may be targeted in COVID − 19, because these cells express receptors for SARS-CoV-2 [18]. In experimental studies, contrast agents reduced renal blood flow and induced oxygen free radicals, a scenario that leads to apoptosis of renal tubular cells [19] and reduced GFR.

Our patients with eGFR 30–60 at the time of contrast-enhanced CT had higher CRP levels and a higher incidence of chronic renal disease compared to patients with eGFR> 60 at the time of contrast-enhanced CT. Severely ill patients with COVID − 19 that have moderate renal dysfunction might be more prone to the effects of iodinated contrast agents, due to the combination of the previous kidney disease and the SARS CoV-2 effect on tubular cells. Severe renal failure is a contra-indication for contrast agent injections; this is the main reason for the high P-creatinine level found in the unenhanced CT group.

Four out of 127 patients in the contrast-enhanced CT group started renal replacement therapy during the study period. Among the patients that were taking renal replacement therapy at the time of the CT scan, normal contrast doses did not cause obvious negative effects, compared to other patients with eGFR≤60 ml/min/1.73 m^2^. However renal replacement therapy and contrast-enhanced CT was associated with CI-AKI at days 4–10 and caution before contrast agent administration for this group of patients seems appropriate.

Our study had some limitations. Firstly, we evaluated relatively small numbers of patients in most of our study groups. Secondly, we lacked follow-up P-creatinine values at 4–10 days in 28% of patients in the eGFR> 60 contrast-enhanced CT group. The reasons for this loss included death before follow-up (*n* = 6), missing follow-up P-creatinine levels, and hospital discharge.

To minimize the risks for CI-AKI we recommend that strict indications are practiced for contrast-enhanced CT in patients with an eGFR≥30–60 ml/min/1.73 m^2^. We propose that iodinated contrast agent, as far as it is possible, should not be administrated to COVID-19 patients with eGFR below 60 protected by full-dose thromboembolic treatment. Accessing pulmonary trunk diameter is an alternative method to diagnose pulmonary thromboembolism through unenhanced CT [20] thereby potentially avoiding the use of contrast agents. Echo cardiac ultrasound is another option to gain information on cardiac dimensions and right ventricular enlargement. One might also speculate that parenchymal abnormalities in the patients [21] are of interest as more widespread infiltrates could correlate with increased risk for LE. If considered necessary contrast-enhanced CT should be performed with a low kV setting and the lowest possible dose of a low- or iso-osmolality contrast agent [22]. In addition, an infusion of isotonic saline, started hours before exposure to contrast agents, has been proposed for protection against CI-AKI [23].

## Conclusion

In conclusion, we demonstrated that hospitalized COVID-19 patients with moderate renal impairment and an eGFR of 30–60 ml/min/1.73 m^2^, showed an increased risk of worsened renal impairment after an iodine-based contrast injection. We observed a > 50% rise in P-creatinine or renal replacement therapy initiation at 4–10 days after contrast-enhanced CT in 36% of patients with eGFR between 30 and 60, compared to 5% of patients with eGFR above 60. Significant differences in CI-AKI incidence between the groups were found, however, as this is a retrospective study larger studies are needed to confirm our observations. This report could aid in risk assessments before ordering a contrast-enhanced CT in patients with COVID-19.

## Data Availability

The datasets generated and/or analysed during the current study are available in the figshare.com repository, DOI: 10.6084/m9.figshare.14658501

## References

[1] Ronco C, Stacul F, McCullough PA (2013). Subclinical acute kidney injury (AKI) due to iodine-based contrast media. Eur Radiol.

[2] van der Molen AJ, Reimer P, Dekkers IA, Bongartz G, Bellin MF, Bertolotto M, Clement O, Heinz-Peer G, Stacul F, Webb JAW, Thomsen HS (2018). Post-contrast acute kidney injury - part 1: definition, clinical features, incidence, role of contrast medium and risk factors : recommendations for updated ESUR contrast medium safety committee guidelines. Eur Radiol.

[3] Thomsen HS (2003). Guidelines for contrast media from the European Society of Urogenital Radiology. AJR Am J Roentgenol.

[4] Hossain MA, Costanzo E, Cosentino J, Patel C, Qaisar H, Singh V, Khan T, Cheng JS, Asif A, Vachharajani TJ (2018). Contrast-induced nephropathy: pathophysiology, risk factors, and prevention. Saudi J Kidney Dis Transpl.

[5] Hoste EA, Doom S, De Waele J, Delrue LJ, Defreyne L, Benoit DD, Decruyenaere J (2011). Epidemiology of contrast-associated acute kidney injury in ICU patients: a retrospective cohort analysis. Intensive Care Med.

[6] Helms J, Tacquard C, Severac F, Leonard-Lorant I, Ohana M, Delabranche X, Merdji H, Clere-Jehl R, Schenck M, Fagot Gandet F, Fafi-Kremer S, Castelain V, Schneider F, Grunebaum L, Angles-Cano E, Sattler L, Mertes PM, Meziani F, Group CT (2020). High risk of thrombosis in patients with severe SARS-CoV-2 infection: a multicenter prospective cohort study. Intensive Care Med.

[7] Wichmann D, Sperhake JP, Lutgehetmann M, Steurer S, Edler C, Heinemann A, Heinrich F, Mushumba H, Kniep I, Schroder AS, Burdelski C, de Heer G, Nierhaus A, Frings D, Pfefferle S, Becker H, Bredereke-Wiedling H, de Weerth A, Paschen HR, Sheikhzadeh-Eggers S, Stang A, Schmiedel S, Bokemeyer C, Addo MM, Aepfelbacher M, Puschel K, Kluge S (2020). Autopsy findings and venous thromboembolism in patients with COVID-19. Ann Intern Med.

[8] Righini M, Robert-Ebadi H (2018). Diagnosis of acute pulmonary embolism. Hamostaseologie.

[9] Kooiman J, Pasha SM, Zondag W, Sijpkens YW, van der Molen AJ, Huisman MV, Dekkers OM (2012). Meta-analysis: serum creatinine changes following contrast enhanced CT imaging. Eur J Radiol.

[10] Barrios Lopez A, Garcia Martinez F, Rodriguez JI, Montero-San-Martin B, Gomez Rioja R, Diez J, Martin-Hervas C (2020). Incidence of contrast-induced nephropathy after a computed tomography scan. Radiologia.

[11] Lautin EM, Freeman NJ, Schoenfeld AH, Bakal CW, Haramati N, Friedman AC, Lautin JL, Braha S, Kadish EG, Sprayregen S (1991). Radiocontrast-associated renal dysfunction: incidence and risk factors. AJR Am J Roentgenol.

[12] McCullough PA, Adam A, Becker CR, Davidson C, Lameire N, Stacul F, Tumlin J, Panel CINCW (2006). Risk prediction of contrast-induced nephropathy. Am J Cardiol.

[13] Ehrmann S, Quartin A, Hobbs BP, Robert-Edan V, Cely C, Bell C, Lyons G, Pham T, Schein R, Geng Y, Lakhal K, Ng CS (2017). Contrast-associated acute kidney injury in the critically ill: systematic review and Bayesian meta-analysis. Intensive Care Med.

[14] Edler C, Schroder AS, Aepfelbacher M, Fitzek A, Heinemann A, Heinrich F, Klein A, Langenwalder F, Lutgehetmann M, Meissner K, Puschel K, Schadler J, Steurer S, Mushumba H, Sperhake JP (2020). Dying with SARS-CoV-2 infection-an autopsy study of the first consecutive 80 cases in Hamburg, Germany. Int J Legal Med.

[15] Kamel MH, Yin W, Zavaro C, Francis JM, Chitalia VC (2020). Hyperthrombotic milieu in COVID-19 patients. Cells.

[16] Nadim MK, Forni LG, Mehta RL, Connor MJ, Liu KD, Ostermann M, Rimmele T, Zarbock A, Bell S, Bihorac A, Cantaluppi V, Hoste E, Husain-Syed F, Germain MJ, Goldstein SL, Gupta S, Joannidis M, Kashani K, Koyner JL, Legrand M, Lumlertgul N, Mohan S, Pannu N, Peng Z, Perez-Fernandez XL, Pickkers P, Prowle J, Reis T, Srisawat N, Tolwani A, Vijayan A, Villa G, Yang L, Ronco C, Kellum JA (2020). COVID-19-associated acute kidney injury: consensus report of the 25th acute disease quality initiative (ADQI) workgroup. Nat Rev Nephrol.

[17] Ackermann M, Verleden SE, Kuehnel M, Haverich A, Welte T, Laenger F, Vanstapel A, Werlein C, Stark H, Tzankov A, Li WW, Li VW, Mentzer SJ, Jonigk D (2020). Pulmonary vascular Endothelialitis, thrombosis, and angiogenesis in Covid-19. N Engl J Med.

[18] Xia S, Wu M, Chen S, Zhang T, Ye L, Liu J, Li H (2020). Long term culture of human kidney proximal tubule epithelial cells maintains lineage functions and serves as an ex vivo model for coronavirus associated kidney injury. Virol Sin.

[19] Bakris GL, Lass N, Gaber AO, Jones JD, Burnett JC (1990). Radiocontrast medium-induced declines in renal function: a role for oxygen free radicals. Am J Phys.

[20] Esposito A, Palmisano A, Toselli M, Vignale D, Cereda A, Rancoita PMV, et al. Chest CT-derived pulmonary artery enlargement at the admission predicts overall survival in COVID-19 patients: insight from 1461 consecutive patients in Italy. Eur Radiol. 2021;31(6):4031–41. 10.1007/s00330-020-07622-x. 10.1007/s00330-020-07622-x PMC7755582 33355697

[21] Aoki R, Iwasawa T, Hagiwara E, Komatsu S, Utsunomiya D, Ogura T (2021). Pulmonary vascular enlargement and lesion extent on computed tomography are correlated with COVID-19 disease severity. Jpn J Radiol Jan.

[22] Mehran R, Dangas GD, Weisbord SD (2019). Contrast-associated acute kidney injury. N Engl J Med.

[23] Fahling M, Seeliger E, Patzak A, Persson PB (2017). Understanding and preventing contrast-induced acute kidney injury. Nat Rev Nephrol.

